# Cerebral Blood Flow Regulation in Pregnancy, Hypertension, and Hypertensive Disorders of Pregnancy

**DOI:** 10.3390/brainsci9090224

**Published:** 2019-09-04

**Authors:** Maria Jones-Muhammad, Junie P. Warrington

**Affiliations:** Department of Neurology, Department of Neurobiology & Anatomical Sciences, University of Mississippi Medical Center, Jackson, MS 39216, USA

**Keywords:** cerebral blood flow, pregnancy, preeclampsia, eclampsia, chronic hypertension, superimposed preeclampsia, blood-brain barrier, autoregulation

## Abstract

The regulation of cerebral blood flow (CBF) allows for the metabolic demands of the brain to be met and for normal brain function including cognition (learning and memory). Regulation of CBF ensures relatively constant blood flow to the brain despite changes in systemic blood pressure, protecting the fragile micro-vessels from damage. CBF regulation is altered in pregnancy and is further altered by hypertension and hypertensive disorders of pregnancy including preeclampsia. The mechanisms contributing to changes in CBF in normal pregnancy, hypertension, and preeclampsia have not been fully elucidated. This review summarizes what is known about changes in CBF regulation during pregnancy, hypertension, and preeclampsia.

## 1. Introduction to Cerebral Blood Flow (CBF) Regulation

**Figure 1:**
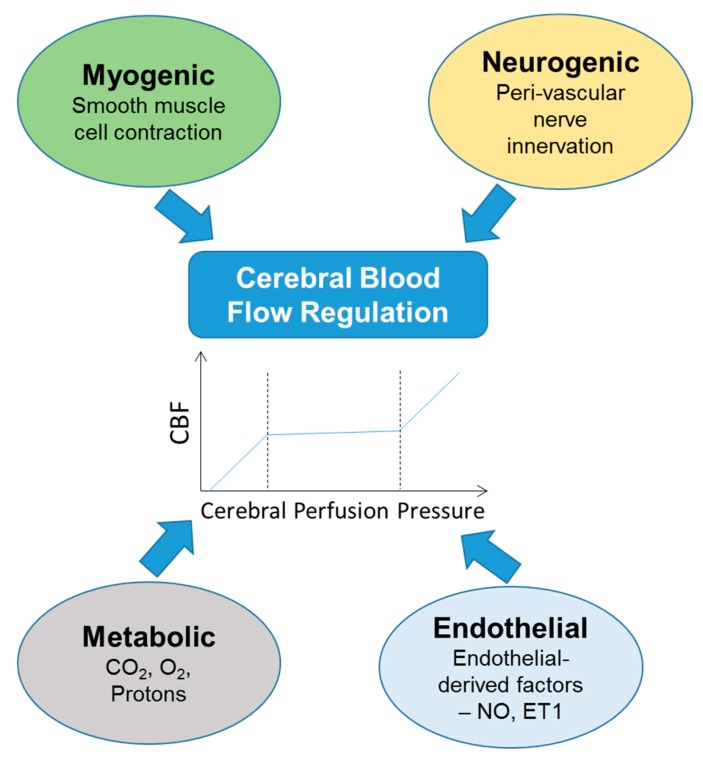
Cerebral blood flow can be regulated by four major mechanisms: myogenic, neurogenic, metabolic, or endothelial. These mechanisms ensure that cerebral blood flow (CBF) is maintained within a relatively normal range. NO—nitric oxide, ET1—endothelin 1.

Cerebral blood flow (CBF) describes the supply of blood to the brain at any moment in time. CBF is tightly regulated to meet the demanding needs of the brain for oxygen, glucose, and other metabolites. Cerebral blood perfusion is maintained relatively constant between mean arterial pressures (MAP) of about 60 and 160 mmHg but rises linearly with blood pressures below or above that range [1]. Although the brain accounts for 2% of our body mass, it is a highly metabolic organ, consuming ~20% of energy at rest. CBF studies rely on different measurement techniques; an excellent review of measurement techniques for CBF and autoregulation can be found here [2]. The arteries involved in supplying blood to cortical areas of the brain are located in the Circle of Willis. The vertebro-basilar system supplies blood to the cerebellum, occipital lobe, and brain stem while the internal carotid arteries supply blood primarily to the cerebrum. Regulated blood flow allows for cerebral tissues to not only get the oxygen needed, but allows for the transport of glucose, various metabolites and other substances across the blood-brain barrier (BBB). CBF regulation can occur through myogenic, neurogenic, metabolic, or endothelial control (Figure 1). Myogenic control induces vascular diameter changes through smooth muscle cell contraction in response to increases in blood pressure. Neurogenic control occurs through perivascular nerves, reviewed here [3,4]. Metabolic control occurs in response to changes in carbon dioxide, oxygen, and protons and is tightly linked to neuronal activity, a process known as functional hyperemia or neurovascular coupling. Endothelial control occurs in response to factors released from endothelial cells such as nitric oxide, which acts as a vasodilator [4] or endothelin-1, a vasoconstrictor.

CBF is thought to be regulated by both extrinsic and intrinsic factors, where the non-penetrating blood vessels at the pia are innervated by extrinsic nerves from the peripheral nervous system while blood vessels that have left the Virchow-Robin space are innervated by intrinsic nerves from the brain neuropil [5]. Regulation of CBF requires the close association of several cell types with the cerebral vasculature, together forming the neurovascular unit. Components of the neurovascular unit include endothelial cells, pericytes, perivascular nerves, smooth muscle cells, and astrocytes [6]. With the close interaction between vessels and neurons, there is a coupling of local blood flow to neuronal activity, known as neurovascular coupling or functional hyperemia. Neurovascular coupling allows for increased blood flow to areas of the brain that are consuming energy due to increased neuronal activity, allowing for homeostasis post-neuronal firing through increased oxygen and nutrient delivery and increased removal of metabolic waste products [6].

CBF autoregulation is classified as static or dynamic based on the method of measurement [3]. Static CBF autoregulation refers to the measurement of the relationship between CBF and MAP, generally at rest (Table 1). Most static CBF autoregulation protocols utilize pharmacological manipulations to induce changes in MAP and an autoregulatory index (ARI) range from 0–1 is generally calculated. An ARI of 0 denotes absent autoregulation, and 1 represents perfect autoregulation [3]. Dynamic CBF autoregulation involves measuring CBF changes in response to rapid changes in MAP and usually measures the time required for CBF to recover to baseline. In the clinical setting, dynamic CBF autoregulation is most commonly assessed. The time taken for CBF to return to normal after changes in MAP can determine dynamic autoregulation through the ARI [3]. Other methods of dynamic CBF autoregulation analysis include neurovascular wavelet analysis [7], transfer function analysis [8], or transient hyperemic response test [9].

Several conditions have been associated with altered CBF autoregulation, including aging, pregnancy, hypertension, and hypertensive disorders of pregnancy. According to a population study investigating CBF in healthy young and healthy elderly patients, CBF is reduced with age, which may be due in part to a reduction in brain volume [10]. The forthcoming sections will discuss evidence for changes that occur in response to hypertension, pregnancy, and hypertensive disorders of pregnancy.

### 1.1. CBF Regulation in the Normotensive and Hypertensive Non-Pregnant State

Hypertension is associated with reduced CBF, which may occur as a result of increased cerebrovascular resistance achieved through vascular remodeling and smaller diameter vessels (reviewed here [11]). When vascular remodeling occurs in a hypertensive environment, cerebral blood vessels are protected from sudden increases in blood flow. While the remodeling process protects the vessels from higher than normal spikes in blood pressure, it can impair the ability to control blood flow at lower blood pressures. Thus, the systems in place to auto-regulate CBF are dysregulated [12]. In a clinical study investigating the impact that untreated chronic hypertension has on CBF, it was found that CBF is significantly reduced in the untreated group or treated group with poor blood pressure control when compared to the treated group with good or intermediate blood pressure control [13]. In older patients with hypertension, blood pressure is increased and CBF is reduced specifically in the left hippocampus, bilateral putamen, Globus pallidus, temporal, frontal, parietal, and orbital frontal regions. This reduction in CBF may result in older patients having a greater risk of developing dementia, or other cognitive impairments [14].

A lot of our understanding of the mechanisms contributing to hypertension-induced changes in CBF has arisen from basic science or pre-clinical studies. To investigate how CBF regulation changes in the hypertensive state, several animal models have been established. In spontaneously hypertensive rat model (SHR), it was found that, compared to the normotensive control group (Wistar-Kyoto rats), there was a significant reduction in cerebral arterial blood volume, but not relative CBF, in the sensory cortex, thalamus, and the hippocampus [15]. Additionally, when dynamic CBF responses to hypercapnia were measured, SHRs had a significantly higher vascular reactivity (the ability of the blood vessels to change their diameter in response to various stimuli) compared to the normotensive control rats. The authors attributed this increased reactivity to the young age of rats studied and proposed that reduced cerebrovascular reactivity is expected with advanced hypertension [15]. Using the same SHR animal model, it was found that the internal carotid artery, middle cerebral artery (MCA), and azygos veins decreased in diameter with age [16] suggesting that prolonged exposure to hypertension contributes to narrowing of the cerebral vessels. Moreover, there was a greater reduction in dynamic CBF response to hypercapnia in neuronal territories supplied by the MCA, anterior cerebral artery (ACA), and the basilar artery [16]. Taken together, studies in the SHR support the conclusion that hypertension induces narrowing of cerebral blood vessels and impairs dynamic CBF response in an exposure duration-dependent manner.

The Dahl salt-sensitive (Dahl-S) rat is another animal model used to study hypertension. One study investigating the impact of hypertension on regional CBF and glucose utilization using the Dahl-S rat found that hypertension had no impact on regional CBF when measured using the hydrogen clearance method [17]. This study, unlike the previously mentioned SHR study, assessed changes in blood flow in the sensorimotor and visual cortex only. The authors attributed the lack of significant changes in CBF to the absence of vascular remodeling in the Dahl-S rats [17]. Another study using the Dahl-S rat found a right-ward shift in static CBF autoregulation with increases in exposure duration to high salt (8.7%) [18].

Another popular model of hypertension is Angiotensin II-induced hypertension. Several studies have utilized this model to understand the effects of hypertension on CBF regulation. Angiotensin II- induced hypertension impairs neural activity-induced increases in CBF in mice [19]. This impaired neurovascular coupling was shown to occur through NADPH signaling [20]. Other studies have shown that in young mice, Angiotensin II extends CBF autoregulation to higher blood pressures while in old mice, CBF autoregulation was disrupted [21]. Importantly, another study showed that dysfunction of the cerebral vasculature occurs before the development of hypertension in the Angiotensin II model of hypertension [22]. Taken together, these studies suggest that hypertension causes structural adaptations of the cerebral arteries that help protect the brain from increases in blood pressure, shifting the auto-regulatory range of CBF in young groups towards higher pressures.

### 1.2. CBF Regulation in Pregnancy and Preeclampsia

Pregnancy is accompanied by major adaptations in response to changes in maternal hemodynamics. Maternal blood volume increases along with cardiac output, with preservation of normal blood pressure (Reviewed in [23]). There is reconfiguration of blood vessels mediated by changes in the plasma levels of vasoconstrictors and vasodilators (nitric oxide), as well as a physical remodeling of the blood vessels [23]. Posterior cerebral artery (PCA) segments from non-pregnant rats constrict significantly more when plasma from pregnant women is added compared to plasma from non-pregnant women [24]. The same study demonstrated that if artery segments from late pregnant rats are exposed to the same plasma from pregnant women, vasoconstriction does not occur. The authors proposed that the lack of vasoconstriction in the late pregnant artery segment may be due to vascular remodeling that may have occurred in pregnancy. The study, however, demonstrated the phenomenon ex vivo rather than in vivo. In a clinical study investigating the changes in CBF velocities in women in the second and third trimester versus non-pregnant women, the authors observed a significant drop in CBF velocity in both pregnancy time-points compared to the non-pregnant group [25]. Additionally, an enhancement of ARI in pregnant versus non-pregnant women was observed, explained partly by the reduced end-tidal carbon dioxide observed in pregnancy [25]. In another clinical study involving normal pregnant women in the first, second, and third trimester, a significant increase in internal carotid artery blood flow volume was observed as the gestational weeks progressed [26]. This increase in CBF was attributed to a decrease in vascular resistance and an increase in the diameter of the internal carotid artery. In another clinical study investigating maternal CBF changes before delivery and within 24 hours postpartum, CBF velocity increased in the middle cerebral artery (MCA) a day after delivery, but this increase was not significant when compared to the non-pregnant control group [27]. Together, these studies demonstrate that in normal pregnancy, CBF velocity decreases, CBF volume increases, and vascular resistance decreases. Within 24 hours of delivery, CBF velocities and cerebovascular resistance return to non-pregnant levels. There is evidence that these adaptations to pregnancy are different in complicated pregnancies.

**Figure 2:**
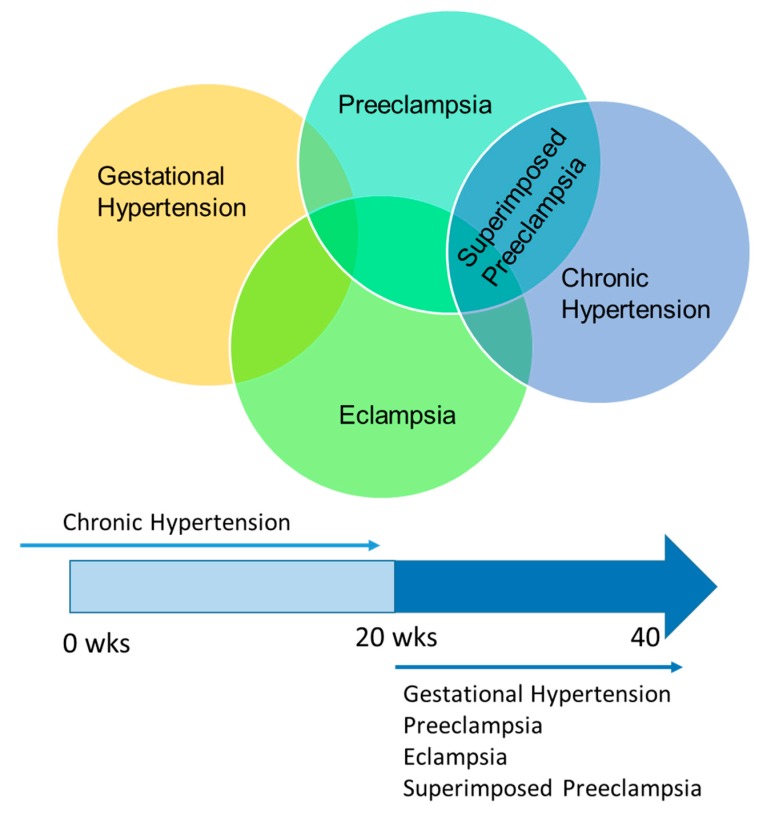
Different hypertensive disorders of pregnancy and their relationship to each other. This diagram gives a visual representation of how the varying pregnancy-associated hypertensive disorders overlap. A subset of women can develop eclampsia from any of the hypertensive disorders of pregnancy and can also develop in normotensive patients. The subset of women with chronic hypertension who develop preeclampsia symptoms are diagnosed with superimposed preeclampsia. Some women with gestational hypertension may develop preeclampsia later in the pregnancy. Only chronic hypertension can be diagnosed before the 20th week of gestation.

While there are other pregnancy complications, for the purpose of this review, we focus only on hypertensive disorders of pregnancy. Hypertensive complications of pregnancy include gestational hypertension, preeclampsia, eclampsia, chronic hypertension, and superimposed preeclampsia. The relationship among these different pregnancy complications is shown in Figure 2.

Gestational hypertension is diagnosed in pregnant women presenting with new-onset hypertension after the 20th week of gestation without other symptoms suggestive of preeclampsia. Preeclampsia is diagnosed when new-onset hypertension occurs after 20 weeks of gestation with one of the following symptoms: proteinuria or complications involving the kidney, platelets, lungs, liver, or brain [28]. Pregnant women, including women with a diagnosis of preeclampsia, chronic hypertension, superimposed preeclampsia, or gestational hypertension, are diagnosed with eclampsia if they have new onset seizures and no prior history of seizure disorder. Some women are hypertensive before becoming pregnant or develop hypertension before the 20th week of gestation and are characterized as having chronic hypertension. If these women develop preeclampsia symptoms, they are diagnosed with superimposed preeclampsia (Figure 2). Several clinical and basic science studies have begun to address the impact of pregnancy and hypertensive disorders of pregnancy on autoregulation of CBF.

Preeclampsia, one of the hypertensive disorders of pregnancy, has been shown to have conflicting effects on CBF. While it is well appreciated that cerebrovascular abnormalities are common complications in preeclampsia, eclampsia, and hemolysis, elevated liver enzymes, low platelet count (HELLP) syndrome [29,30,31,32,33,34], some studies have reported increased cerebral perfusion pressure while others have reported reduced cerebral perfusion pressure in preeclampsia patients. In a case-control study conducted by van Veen et al., women with preeclampsia had impaired dynamic CBF, a higher resistance index, increased cerebral perfusion pressure, and an overall lower dynamic CBF auto-regulatory index than the control, normal pregnant group [35].

**Figure 3:**
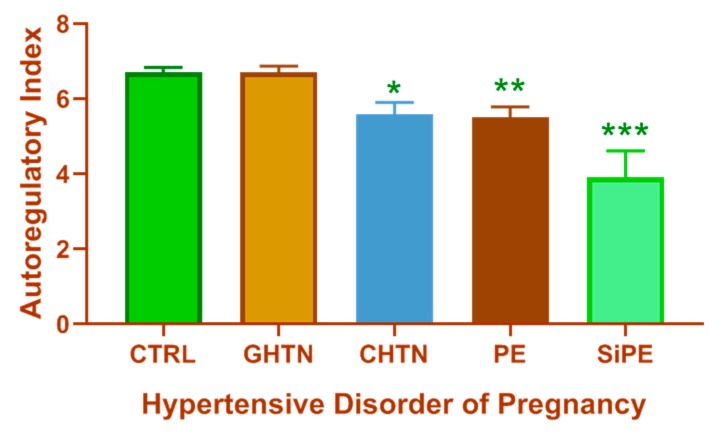
Dynamic CBF autoregulation differs among the different hypertensive disorders of pregnancy. Compared to normal pregnant women (CTRL), pregnant women with chronic hypertension (CHTN), preeclampsia (PE) or superimposed preeclampsia (SiPE) have significantly lower autoregulatory index during pregnancy while women with gestational hypertension (GHTN) have an autoregulatory index similar to that of the CTRL. Women with superimposed preeclampsia have the lowest autoregulatory index of all hypertensive disorders of pregnancy. * *p* < 0.05, ** *p* < 0.01, *** *p* < 0.001 compared to CTRL. This figure was created using data presented in van Veen, et al. AJOG, 2015 [36].

Another study assessing differences in cerebral perfusion pressure in patients with chronic hypertension during pregnancy or superimposed preeclampsia showed that women with superimposed preeclampsia had increased cerebral perfusion pressures and greater vascular resistance compared to those with chronic hypertension [37]. Furthermore, increased cerebral perfusion pressure in the anterior and posterior cerebral arteries [38], increased CBF velocities in the MCAs [30,39], along with reduced dynamic CBF autoregulation have been reported in preeclampsia patients when compared to normal pregnant women [35]. Cortical and/or subcortical lesions involving the occipital lobe have been reported in preeclampsia and eclampsia patients [39] or patients diagnosed with eclamptic encephalopathy [40]. Figure 3 summarizes studies from The Netherlands where the effect of spontaneous changes in blood pressure on CBF velocities from different hypertensive pregnancy groups were assessed [36]. As shown, women with superimposed preeclampsia have the most impaired dynamic CBF autoregulation of all the hypertensive disorders of pregnancy with significantly lower autoregulatory index compared to all other groups.

The impact of eclampsia on CBF has been addressed in some case studies and other small studies with conflicting findings reported. For example, in postpartum eclampsia cases (where eclampsia is diagnosed after delivery of the baby), hyperperfusion in parietal and occipital white matter [41], increased CBF velocity in all cerebral vessels [42] and even decreased local CBF measured using xenon/computed tomography in ACA and PCAs [43] have been reported. Other studies have shown that compared to patients with severe preeclampsia, patients with eclampsia had increased CBF velocities and further increases in CBF velocities in the post-seizure phase [44]. Another study of two eclampsia cases found no sign of cerebral vasospasm but increased cerebral glucose metabolism suggesting increased neuronal activity in eclampsia patients [45]. Taken together, these studies demonstrate that in majority of cases, CBF velocities increase in eclampsia patients. It should be noted that changes in CBF velocity as measured via transcranial Doppler, does not fully reflect CBF which takes into account changes in vessel diameter, an unknown parameter in transcranial Doppler measurements.

Not only is preeclampsia associated with impaired CBF regulation during pregnancy, but CBF may be changed in the postpartum period as well. Indeed, women who had preeclampsia in a previous pregnancy are reported to have worse dynamic CBF autoregulation compared to those who had normal prior pregnancies [46]. Furthermore, women with a history of preeclampsia have reduced cerebrovascular reactivity compared to women with a history of a normotensive pregnancy [47]. Taken together, there is evidence that CBF regulation is impaired in patients with preeclampsia during pregnancy as well as in the postpartum period. Studies are now beginning to elucidate the mechanisms contributing to this dysregulation of CBF associated with preeclampsia and other hypertensive disorders of pregnancy.

### 1.3. Consequences of Impaired CBF Regulation

Impaired CBF autoregulation can have several detrimental effects. One of the consequences of impaired dynamic CBF autoregulation is increased BBB permeability, which can lead to cerebral edema (reviewed in [48,49]). Failure of cerebral blood vessels to auto-regulate in response to increases in blood pressure leads to increased hydrostatic pressure, potentially damaging the micro-vessels, resulting in increased BBB permeability, micro-bleeds, neuroinflammation, and neuronal damage. Chronically, neuronal degeneration, capillary rarefaction, and subsequent functional deficits may result [50]. The prevailing hypothesis is that CBF that is not controlled within the auto-regulatory range can result in impaired cognition as well as impaired overall neuronal function [51], which may increase the chance of developing vascular dementia or Alzheimer’s disease.

There is a growing body of evidence supporting the hypothesis that a history of hypertensive disorders of pregnancy increases the risk of long-term neurovascular abnormalities. For example, in a retrospective cohort study, a history of hypertensive disorders of pregnancy was associated with an increased risk of mortality from Alzheimer’s disease and stroke years after index pregnancy [52]. A prospective study conducted in Sweden found that women with a history of preeclampsia experienced their first stroke earlier than women with a history of gestational hypertension or normotensive pregnancies with no impact on the incidence of dementia [53]. One limitation of that study was that participants were required to have intact cognitive function in order to be included in the study; thus, women with early onset dementia were missed. Importantly, a recent Danish study showed that women with a history of preeclampsia (preeclampsia, eclampsia, or HELLP syndrome) had a significant increase in vascular dementia diagnosis [54]. Furthermore, women with a history of (pre)eclampsia had an 80% increased risk of stroke when compared to women who had a normotensive pregnancy history [55]. Moreover, women with a history of preeclampsia reported higher cognitive difficulties in their daily lives, performed worse on cognitive tasks [56,57], and were more likely to have white matter lesions observed as hyper-intense areas on brain imaging [58] when compared to women with normal pregnancy history. Taken together, compelling evidence suggests that preeclampsia does not end at delivery but is associated with increased risk for several long-term consequences for the affected mother. It should be noted that the evidence for decline in cognitive function were based on subjective rather than objective endpoints.

In addition to the mother, preeclampsia can have major consequences on the health of the offspring as well. There is growing evidence suggesting that offspring exposed to preeclampsia in utero are at increased risk of neurological morbidities later in life [59]. A study investigating the difference in neural anatomy of children born to mothers with preeclampsia versus those without showed that children exposed to preeclampsia had enlarged neural areas such as the amygdala, cerebellum, temporal lobe, and brain stem [60]. They further suggest that these differences may be attributable to reduced levels of placental growth factor (PlGF), which is a vasodilator similar to vascular endothelial growth factor (VEGF).

In the mother, if CBF dysregulation persists, BBB leakage and cerebral edema may occur. The current hypothesis is that increased BBB permeability and cerebral edema increases the risk of cognitive dysfunction. A clinical study investigating the impact that increased BBB permeability may have on cognition concluded that individuals with Alzheimer’s disease had increased BBB permeability and a reduction in the hippocampal area; however, the study did not determine whether vascular dementia increased this permeability [61].

### 1.4. Limitations of Current Knowledge of CBF Regulation in Hypertensive Disorders of Pregnancy

Several animal models have been developed to further investigate the impact of pregnancy and hypertensive disorders of pregnancy on CBF. Table 2 summarizes some of the animal models of pregnancy and experimental preeclampsia in which aspects of CBF function were assessed.

One animal model of preeclampsia is the reduced uterine perfusion pressure model (RUPP), which mimics the reduction in utero-placental blood flow reported in preeclampsia patients [74]. Using the RUPP model in rats, myogenic response in isolated MCA segments was impaired under active (with calcium) versus passive (without calcium) conditions. In the pregnant control and non-pregnant control groups, the vessel diameter decreased in the active state compared to the passive state; however, the diameter did not change as much in the RUPP model, indicating that its ability to respond to increases in blood pressure is impaired [62]. This impaired MCA myogenic reactivity was associated with increased brain water content in the RUPP model compared to the non-pregnant and normal pregnant groups [62]. These ex vivo findings were then followed up in vivo, where it was shown that the impaired myogenic reactivity of isolated cerebral vessels was associated with impaired static CBF autoregulation [64]. The authors concluded that since there was no evidence of vascular remodeling in the RUPP, the impairment of CBF autoregulation most likely occurred because vascular remodeling had not taken place. Furthermore, both brain water content and BBB permeability increased in placental ischemic rats [64]. Other studies have shown that when hypertension is induced after the initiation of pregnancy, hypertension-induced cerebrovascular remodeling does not occur [68,69,70]. Moreover, a related study showed that pregnancy reverses hypertension-induced cerebrovascular remodeling [70]. Other studies suggest that during pregnancy, increased CBF caused by forced dilation of cerebral vessels occurs at lower blood pressures [68,69,75,76], although the studies used normotensive late pregnant rats in which blood pressure was raised acutely. Additionally, an animal study investigating the impact exposure to placental ischemia may have in the postpartum period demonstrated that two months after delivery, rats with a history of placental ischemia had increased brain water content in the posterior cerebral cortex, which may be due to increased BBB permeability [63].

Looking further into how preeclampsia may impact CBF, another study found that when plasma from women with preeclampsia was applied to cerebral veins of late pregnant rats, there was an increase in hydraulic conductance (permeability) of the blood vessels with no change in vascular reactivity or tone [77]. The increase in vascular permeability was prevented by addition of vascular endothelial growth factor receptor (VEGFR2) to preeclampsia plasma or VEGF without plasma, which demonstrated that increased cerebrovascular permeability in preeclampsia may be through increased activity of the VEGF receptor tyrosine kinase [77].

Several groups have begun to assess the neurological complications of eclampsia using various animal models. In these animal models, seizures are generally induced in pregnant animals or in animal models of preeclampsia. For example, using a model of severe preeclampsia where placental ischemic rats are fed high cholesterol diet, no difference in relative CBF was found. When these rats were treated with pentylenetetrazol (PTZ) to induce seizures, the authors reported an increase in CBF in non-pregnant and pregnant rats with no change in the preeclampsia group during seizure activity [66]. Other studies have induced seizures in the RUPP rat [72,78] and LPS-injection model but did not assess CBF parameters in the studies. More studies are needed to address the mechanisms underlying seizures in eclampsia, and to delineate the CBF responses before, during, and after seizure experience.

The Dahl- S rat, in addition to being used as a model of hypertension, is also used as a model of preeclampsia, specifically, superimposed preeclampsia. Dahl- S rats are hypertensive and have exacerbated proteinuria in mid and late pregnancy when maintained on a normal salt diet compared to virgin rats [79]. The cerebrovascular changes that occur in the Dahl-S model of superimposed PE have not yet been described. Another preeclampsia model is a genetic mouse model (endothelial nitric oxide synthase (eNOS)^−/−^ mouse model) where endothelial nitric oxide synthase is knocked out. These mice display symptoms of preeclampsia such as hypertension during pregnancy, proteinuria, restricted fetal growth, and placental hypoxia [80]. When investigating whether these mice display CBF dysfunction, it was found that the knockout mice had reduced blood flow to the caudate putamen and the parietal cortex when nitric oxide synthase is knocked out in endothelial tissue versus neuronal tissue [81].

While there are several different models proposed to mimic the symptoms of various hypertensive disorders of pregnancy, there are no animal models that mimic every characteristic symptom of the different disorders. This limitation is compounded further given that preeclampsia diagnosis can be made using so many different combinations of symptoms. Only hypertension is fixed in the diagnosis of preeclampsia. Despite these limitations, by modeling different aspects of the pregnancy disorder, advances have been made in understanding some of the underlying mechanisms. Another limitation of the current body of literature is the variability in methodology. Parameters of CBF regulation are measured using different techniques (magnetic resonance imaging (MRI), transcranial Doppler, among other methodologies) in the clinic while animal studies also use varied methodologies. Additionally, while the majority of clinical studies assess dynamic CBF autoregulation, majority of animal studies measure static CBF autoregulation. It is clear that methodologies need to be better aligned to improve the translation of animal findings to the clinic. 

## 2. Conclusion and Perspectives

**Figure 4:**
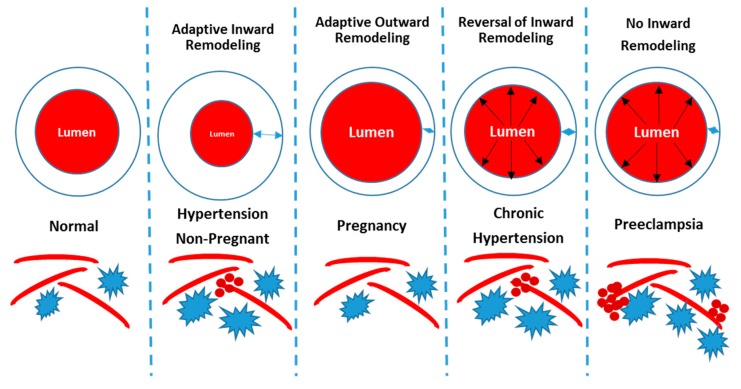
Summary of cerebrovascular changes associated with hypertension, pregnancy, and preeclampsia. In the hypertensive, non-pregnant state, wall thickness increases and lumen diameter decreases. In pregnancy, there is an adaptive outward remodeling while in preeclampsia, there is a lack of inward remodeling in response to hypertension, causing cerebral blood vessels to be more susceptible to blood brain barrier (BBB) disruption and micro-bleeds. In chronic hypertension, pregnancy reverses the in-ward remodeling of the cerebral vessels. Increased blood pressure and velocities in vessels with thin walls can cause transmittal of pressure to the micro-vessels causing BBB leakage and micro-bleeds. This induces increases in glial cells and chronically, neuroinflammation. Tube-like structures represent capillaries, star-shaped cells represent glia, and red mini-circles represent micro-bleeds.

Although there are animal models that allow more in-depth understanding of the impact that pregnancy, hypertension, and preeclampsia have on cerebrovascular function, no animal model is able to display all of the symptoms of preeclampsia. Thus, better animal models are required to enable more insight into the underlying mechanisms. As shown in Figure 4 and discussed earlier, hypertension, pregnancy, chronic hypertension, and preeclampsia induce different structural changes in the cerebral vasculature. Hypertension induces increased wall thickness and smaller lumen, while pregnancy is associated with thinner vascular walls and larger lumen. In preeclampsia, wall thickening does not occur in response to hypertension, which can result in impaired CBF autoregulation, BBB disruption, cerebral edema, and long-term neurological complications postpartum. Pregnancy has been considered a stress test for women [82,83], and there is the notion that women who go on to develop preeclampsia had existing sub-clinical vascular abnormalities that are exacerbated with pregnancy. This hypothesis, although attractive, has not been tested empirically and is quite difficult to test in the clinic. Another hypothesis is that placental factors are secreted as a result of reduced blood flow to the utero-placental unit, initiating the symptoms of preeclampsia, including cerebral symptoms. This hypothesis is being investigated by our and other groups. One of the unanswered questions in the field is centered on why some women with hypertensive disorders of pregnancy develop cerebral symptoms and others do not.

Because of emerging evidence of increased risk of long-term cerebral complications following a hypertensive disorders of pregnancy, longitudinal studies spanning the non-pregnant state to the postpartum period are needed. For these longitudinal studies, structural and functional changes of the cerebral vasculature should be recorded. Additionally, studies geared at developing preventive and treatment strategies are necessary since currently, delivery of the fetus is the only “cure” for preeclampsia. Early delivery, in addition to increasing the number of premature births, comes with increased risks of complications associated with prematurity. Currently, approximately 5% of pregnant women develop preeclampsia, and cerebrovascular complications is a cause of death in ~40% of preeclampsia/eclampsia cases [84]. More studies geared at understanding the underlying pathophysiological mechanisms are warranted since long-term complications for the mother and offspring present a severe healthcare burden to society.

## Figures and Tables

**Table 1:** Comparison between static versus dynamic CBF autoregulation.

	Static CBF Autoregulation	Dynamic CBF Autoregulation
What does it measure?	Steady-state CBF/MAP relationship	Transient changes in CBF in response to rapid MAP changes (CBF recovery time)
Autoregulatory Index range	0–1 0 = absent autoregulation 1 = perfect autoregulation	0–9 0 = absent autoregulation 9 = perfect autoregulation
Methods used to manipulate MAP	Pharmacological agents	Valsalva maneuver Lower body negative pressure Sudden posture changes Thigh-cuff deflation Rapid changes in head positioning Cold pressor stimulus Spontaneous fluctuations in MAP at rest
Type of studies predominantly used	Animal Studies	Clinical Studies

CBF—cerebral blood flow, MAP—mean arterial pressure.

**Table 2:** Summary of animal studies describing the impact of pregnancy or pregnancy complicated by hypertension on CBF.

Condition Mimicked	Manipulation	Vascular Bed	CBF Response Type	CBF-Related Response	Ref.
PE	RUPP	MCA segments	Myogenic Tone	Impaired Myogenic tone in isolated vessels	[62,63]
PE	RUPP	MCA territory	Static CBF	Impaired CBF autoregulation	[64]
PE	High Cholesterol	Cortical Parenchymal Arterioles	Myogenic Tone	No change in myogenic tone. ↓ Vasodilation in PE group.	[65]
PE & Eclampsia	High Cholesterol + PTZ	Hippocampal Arterioles	CBF	↑ CBF in non-pregnant and pregnant group but no change in PE group during seizure ↓ Basal tone and ↓ vasodilation in PE group.	[66]
PE & Eclampsia	RUPP + High Cholesterol + PTZ	PCA Territory	Relative CBF	No difference in relative CBF	[67]
Pregnancy	Late Pregnancy	PCA Territory	Static CBF	↑ Vasodilation in pregnant group. CBF auto regulatory curve shifted to lower pressures.	[68]
PE	Acute Phenylephrine Infusion	Anterior + Posterior Cerebrum	Static CBF	Pregnancy ↑ CBF autoregulation threshold in both regions. Acute hypertension ↑ edema in both regions	[69]
Chronic Hypertension	L-NAME (2wks before + during gestation)	PCA segments	Vascular Remodeling	Hypertension = inward remodeling Hypertension + Pregnancy = no inward remodeling	[70]
PE	LPS (1.5 µg/kg)	PCA	Myogenic Tone	↓ Myogenic tone in late pregnant LPS rats	[71]
Eclampsia	RUPP + PTZ	Global	Brain Water Content	↑ Water content with seizures in normal pregnant and RUPP rats	[72]
Eclampsia	Prenancy + PTZ	Posterior Cerebrum	Brain Water Content	↑ Water content in pregnant rats following seizures	[73]

RUPP—reduced uterine perfusion pressure, LPS—lipopolysaccharide, MCA—middle cerebral artery, PCA—posterior cerebral artery, PE—preeclampsia, PTZ—pentylenetetrazol, L-NAME—N(ω)-nitro-L-arginine methyl ester.
